# The Impact of Mild Frost Occurring at Different Harvesting Times on the Volatile and Phenolic Composition of Virgin Olive Oil

**DOI:** 10.3390/antiox11050852

**Published:** 2022-04-27

**Authors:** Catalina Pino, Betsabet Sepúlveda, Francisco Tapia, Jorge Saavedra, Diego L. García-González, Nalda Romero

**Affiliations:** 1 Departamento de Ciencia de los Alimentos y Tecnología Química, Facultad de Ciencias Químicas y Farmacéuticas, Universidad de Chile, Santiago 8380000, Chile; catalina.pino.m@ug.uchile.cl; 2 Centro Para el Desarrollo de la Química—CEPEDEQ, Facultad de Ciencias Químicas y Farmacéuticas, Universidad de Chile, Santiago 8380000, Chile; bsepulveda@ciq.uchile.cl; 3 Instituto de Investigaciones Agropecuarias (INIA Intihuasi), La Serena 1700000, Chile; fatapiac@inia.cl; 4 Escuela de Alimentos, Pontificia Universidad Católica de Valparaíso, Valparaíso 2340000, Chile; jorge.saavedra@pucv.cl; 5 Instituto de la Grasa (CSIC), Campus Universidad Pablo de Olavide, Edificio 46, Ctra. de Utrera, km. 1, 41013 Sevilla, Spain; dlgarcia@ig.csic.es

**Keywords:** olive oil, frost, quality, phenols, volatile compounds

## Abstract

The organoleptic characteristics of virgin olive oil (VOO), together with its nutritional and health properties, have led the olive tree to be cultivated beyond the Mediterranean basin, reaching latitudes with colder climates, with minimum temperatures below 0 °C and with a higher probability of early frosts. The freezing of olives generates destruction within the tissues and promotes degradation reactions. In this study, the impact of mild frost occurring at different harvesting times on the composition of volatiles and phenolic compounds in VOO were investigated. Arbequina variety olives were harvested at different stages of ripening. Half of the olives were subjected to oil extraction and the other half were frozen at −3 ± 1 °C for 12 h prior to oil extraction. A significant decrease of phenolic compounds with harvesting time was observed in both types of oils (fresh and frozen olives). Oils from frozen olives presented a slightly higher content of total phenols, except in the advanced ripening stage (September), and a slightly lower content of volatile compounds at all harvesting times. In addition, a higher content of 3,4-DHPEA-EDA was observed in oils from frozen olives, which is attributed to an early action of the endogenous β-glucosidase enzyme on oleuropein in freeze-damaged olive fruits. Principal component analysis and Discriminant Partial Least Square Regression allowed the oils to be classified according to the type of fruit (fresh and frozen) and the month of harvest. This study would indicate that mild frost would have a low impact on the chemical composition of virgin olive oil, although, this depends on the ripening stage.

## 1. Introduction

The olive tree has a fruit that must be processed in two ways to be consumed, one through microbiological treatments (table olives), and another through physical processes that allow the extraction of the virgin olive oil (VOO) that is present in the pulp, which represents between 59.5% and 32.2% of the fruit on a dry matter basis [1]. The origin of this crop is located on the margins of the Mediterranean Sea [2,3,4], whose agroclimatic conditions of marked seasons allow a slow development from flowering to harvest, which takes 6 to 7 months [5]. In this area, the agroclimatic conditions are characterized by a Mediterranean climate, with seasons well marked by a rainy and cold period from mid-autumn, when temperatures drop from 25 °C to −3 °C, to rise above 20 °C, until mid-spring. Where the rain is scarcer, this season is followed by a dry and warm period, with summer temperatures that exceed 35 °C, and then the temperature slowly drops and reaches less than 0 °C in the middle of winter, completing the cycle. During the autumn period before the onset of frost, the maturity of the fruit is completed and it is harvested before it is damaged by low temperatures [6], to later transform it into an edible product with a high nutritional and functional value [7].

Ideal climatic conditions allow the production of maximum quality oil, maintaining a certain stability in the chemical composition and sensory characteristics, which ensures the uniformity and quality of the oil year after year. The study of the possible ranges in chemical, physical–chemical, and sensory characteristics of the oil between regions, cultivars, and crop seasons has made it possible to establish quality, purity, and nutritional standards for the production of olive oil [8]. The limits and ranges for different parameters defining the different categories of virgin olive oil were initially defined when most of the olive trees were cultivated in the Mediterranean basin [9,10]; however, when the chemical composition of virgin olive oils from different geographical origins is cross-tabulated for comparative purposes, the effect of pedoclimatic conditions on chemical composition is evidenced in the case of oils from the same cultivars obtained in two different locations. In fact, the effect of climate on olive composition has been previously reported since the 90s [11,12,13], and the effect of climate on composition partially forms the basis for other recent studies that are based on the identification of geographical provenance [4,14,15,16].

The production of virgin olive oil outside of the Mediterranean basin has increased considerably in the last years [17]. In fact, the diffusion of the favorable nutritional and functional characteristics of olive oil, together with scientific evidence proving their healthy properties [18], has promoted olive cultivation throughout much of the world [4]. Consequently, new plantations have been developed in different latitudes, whose climatic conditions differ from those existing in the area of origin, incorporating particular agronomic, irrigation, and fertilization managements. In addition, new olive groves have been configured with high planting densities, making this an intensive crop. In particular, new productive poles have been established in different countries of South America, the United States, Australia, South Africa, and China [4,19]. Some of these new olive groves have been developed in colder latitudes where thermal accumulation is lower, requiring a longer maturation time to achieve a full harvest. In fact, the risk of frost is high in some of these latitudes.

Frost starts when the temperature drops below 0 °C, leading to external fruit damage, and it can be severe if temperatures drop below −1.7 °C. In the latter case, freezing damage occurs, and the temperature may accelerate some ripening characteristics, such as softening of the pulp, increasing of olive respiration, production of ethylene, and the increase of pectic enzyme activities [20,21]. In fact, when frost takes place, some physiological changes take place in the tree [22]. There are evergreen plants that have adaptations to different environmental conditions, and this adaptation slowly evolves each season. The tolerance to low temperatures is directly correlated with hardening [23]; however, this hardening is lost when temperatures change sharply from cold to warm [24]. In olive trees, it has been observed that olives that are affected by frost damage and are processed quickly, which may keep the quality indexes within virgin olive oil quality, but the unsaponifiable components such as pigments and phenolic components are affected, and this has an effect of the sensory characteristics [25]. Furthermore, frost generates a destruction of the olive tissues and promotes degradation reactions with a consequent impact upon sensory characteristics [26].

The physiological and chemical changes associated with frosted olives lead to oils that are softer, and have less pungency and bitterness [25]. Furthermore, the change of sensory characteristics may result in a sensory defect defined as ‘frostbitten olives’ by the International Olive Council [27]. Thus, Romero et al. [26,28] proved that there were two types of sensory profiles related with the sensory defect associated to frost. One of these profiles is described as having “soapy” and “strawberry-like” sensory notes, whereas the other was described as having “wood” and “humidity” notes. The different profiles were explained by the different patterns in temperature drops, and several freeze−thaw cycles vs. a gradual drop in temperature.

The chemical and sensory changes exerted by frost depends on many factors, the cultivar being a relevant one since the adaptation to extreme temperatures depends on the genotype of the olive tree [29]. Thus, evaluations carried out in Wudu, China, latitude 33° N, at 1033 m above sea level, showed that the most widespread variety in the world, Arbequina, presented a better adaptation to extreme temperature conditions compared with other seven cultivars, reaching temperatures below −7 °C [30]. Centeno et al. [31] also studied the adaptation of cultivars to cold climates, and they concluded that Arbequina, together with Koroneiki and Sikitita, could be recommended for such climates, taking into account agronomic variables; however, it is evident that when severe frost takes place, for example, maximum temperatures −5 °C for more than 110 consecutive hours, including even temperatures of −12.5 °C, the quality and chemical composition of oils from the Arbequina cultivar is also affected [25].

Virgin olive oils from the Arbequina cultivar has been particularly studied, considering its different climate and weather conditions, and given that this country presents a wide variety of climates, it has been regarded as a geographical location to test climate-composition relationships [9]. Thus, a previous work has been focused upon for the comparison of the composition of functional components and quality of oils obtained from the Arbequina cultivar grown at two different latitudes in Chile [14]. In this work, two climatic zones were evaluated, one located at latitude 30° S, characterized by a climate with a semi-desert influence, and a second located at latitude 35° S, with a greater presence of rains, cold winters, and markedly hot and short summers. The zone of higher latitude does not achieve full maturity, presenting a lower content of phenolic compounds, but higher aromatic compounds compared with the zone of higher solar radiation. The effect of frost on the fruit was not detected by the analysis carried out, with both oils presenting satisfactory levels of quality [14]; however, similarly to many other places, frost is one of the climatological phenomena that needs to be considered in Chile, since it affects operating costs [32].

In the context of a better understanding of the changes that occur when virgin olive oil is obtained from frosted olives, this work is aimed at studying the volatile and phenolic composition in oil from an experimental design perspective, in which temperature is controlled. Attention was especially paid to mild frost (−3 °C), where olive tissues are not so damaged and the changes in phenols and volatile compounds may be less evident, and the ripeness of the product may have an important effect on the result. Furthermore, as climatic conditions are becoming increasingly changeable, with early frosts occurring at different periods of olive growth, the aim of this work was to study the impact of frost at different harvesting times, and investigate how this affects the quality and the composition of the virgin olive oil Arbequina, with its minor components being phenolic and volatile compounds.

## 2. Materials and Methods

### 2.1. Reagents

All reagents, analytical (ethanol, phosphoric acid, isopropanol, ethyl acetate, sodium hydroxide, sodium thiosulfate), HPLC (hexane, methanol, acetonitrile,), or spectroscopy (isooctane) grade were acquired from Merck (Darmstadt, Germany). All standards were identified, as were internal standards, to quantify the phenols and reagents to determine oxygen radical absorbance capacity (ORAC) [33], which were obtained from Sigma-Aldrich (St. Louis, MO, USA). All standards, including the internal standard to identify and quantify volatile compounds [33], were purchased from Merck. The alpha tocopherol standard was acquired from Calbiochem (Darmstadt, Germany).

### 2.2. Orchard Characteristics and Agricultural Aspects

The present study was carried out during the 2019–2020 campaign, in a 20-year-old olive grove of the Arbequina variety, planted in high density (1300 trees per hectare), and located in the INIA Huasco Experimental Center (28°34′43″ S and 70°47′56″ W) at 447 m above sea level. The climatic conditions are Marine Subtropical with a prolonged dry season, winter rainfall of 30 mm, and maximum and minimum temperatures between 28 °C (summer) and 15 °C (winter). Absolute temperatures exceed 30 °C and can reach −7 °C. The soil conditions are old calcareous alluvial plains, 0.3 to 0.5 m deep, with sandy loam to clay loam textures, and a pH between 7.2 and 8.2. The olive trees were irrigated daily according to the reference evapotranspirative demand (ETo), with 5126 m^3^/ha year.

### 2.3. Sample Selection and Processing

Two random rows of trees (15 per row) with similar vigor, age, production, and agronomic management were selected. Five harvesting times between May and September (one by month) were selected, with levels of ripeness between 2–3 and 6 [34]. At each harvest, 15 kg of olives were collected from the middle portion of three trees, and were randomly selected; the olives were mixed and divided into two portions of 7.5 kg each (experimental unit). One portion was subjected to oil extraction. The second portion was stored at −3 ± 1 °C for 12 h in a Freezer Z300 cold chamber (Fensa, Santiago, Chile), then thawed at room temperature for 2 to 4 h, and later subjected to oil extraction. Each harvesting time was collected in triplicate. Only healthy fruits, without any sign of infection or physical damage, were used. In total, 30 samples of VOO were processed.

### 2.4. Olive Oil Extraction

Olives were processed using Frantoino model Monoblock extraction equipment (Toscana Enologica Mori, Firenze, Italy) with a two-phase centrifugation system. Both types of fruits, fresh and frozen olives (7.5 kg), were grinded and then slowly mixed for 30 min at 25 ± 2 °C. The resulting paste was centrifuged at 1027× *g* for 5 min to separate the oil. All samples were later filtered through hydrophilic cotton, placed in amber glass bottles, and stored in the dark at −23 °C until analysis (within 1 month). The samples were analyzed in triplicate using the chemical analytical methods described below.

### 2.5. Quality Parameters

Quality parameters were determined according to AOCS standard methods which were [35] (1993): free fatty acids (Ca 5a-40), peroxide value (Cd 8-53), and specific extinctions of oils (K232, K270) (Ch 5-91).

### 2.6. Color

Color was measured using a Lovibond Tintometer PFX195 instrument (Tintometer Inc., Sarasota, FL, USA). The following color scale parameters were used: CIELAB values L*, a*, and b*; observer/illuminant 10°, and D65, and a path length of 1.5 cm according to UNE-EN ISO/CIE 11664-4:2020 [36]. Shortly afterwards, 10 mL olive oil were placed in a cuvette and the color parameters L*, a* and b* were measured and recorded. The color difference (ΔE) was calculated as follows: ΔE = (ΔL^2^ + Δa^2^ + Δb^2^)^1/2^

### 2.7. Determination of Phenolic Compounds

The phenols were extracted by solid-phase extraction according to Caipo et al. [33] and analyzed in a Waters HPLC system consisting of a binary pump (model 1525), a diode array UV detector (model 2998), and an autosampler (model 2707) coupled to a Waters Spherisorb ODS RP-18 column (4.6 mm i.d. × 250 mm; 5 μm particle size). Simple phenols were identified with Sigma standards, and secoiridoid compounds were identified by comparison of their absorbance spectra with literature data [37,38]. Internal standard p-hydroxyphenylacetic acid was used for the quantification of simple phenols, phenolic acids (other than ferulic acid) and secoiridoid compounds at 280 nm and elenolic acid at 235 nm. Additionally, flavones (luteolin, methyl luteolin and apigenin) were quantified using o-coumaric acid as an internal standard at 335 nm. The results were expressed in mg/kg.

### 2.8. Total Phenolic Content

Total phenols were obtained by liquid/liquid extraction with methanol/water (80/20) according to IOC [37], with modifications according to Fuentes et al. [39] The total phenolic content was quantified spectrophotometrically following the Folin–Ciocalteau colourimetric method. The calibration curve was constructed using six different concentrations of standard solutions of caffeic acid (Sigma Chemicals Co., San Luis, CA, USA), from 50 to 500 μg/mL in triplicate (R^2^ = 0.9983).

### 2.9. Hydrophilic Orac Assay (H-ORACFL)

Antioxidant capacity was determined using the oxygen radical antioxidant capacity method, which uses fluorescein as the fluorescent molecule and a 0.075 M phosphate buffer (pH 7.4) for the preparation of Trolox standard solutions and samples, according to Prior et al. [40] with some modifications, and as was explained in detail by Fuentes et al. [39] The reactions were carried out in a black 96-well plate. An aliquot (25 μL) of the phenolic extract diluted in methanol/water, or 25 uL of Trolox calibration solutions (12.5, 25, 50, and 100 μM), and 150 μL of fluorescein solution were added to each well. After tempering the plate (30 min at 37 °C) the reactions were initiated with the addition of 25 μL (150 mM) of AAPH (2,2′-azobis(2-amidino-propane) dihydrochloride); readings were achieved on a FLx800-TBID fluorescence reader (Biotek, Winooski, VT, USA), at excitation (485 nm)/emission (528) from the top of the plate. Antioxidant capacity was expressed as μmol Trolox equivalent (TE)/g oil.

### 2.10. Tocopherol Content

Quantification of α-tocopherol was evaluated according to AOCS Ce 8-89 (1993) [35] as described by Fuentes et al. [39] using an HPLC consisting of a Merck-Hitachi pump L-6200A (Merck, Darmstadt, Germany), a Rheodyne 7725i injector with 20 μL sample loop, a LiChro-CART Superspher Si 60 column (25 cm × 4 mm id, 5 μm particle size; Merck, Darmstadt, Germany), a Hitachi Chromaster 5440 fluorescence detector, and a PC with Clarity chromatographic software (DataApex, Prague, The Czech Republic) to process the chromatographic signal detected at 290 nm and 330 nm, and the excitation and emission wavelengths, respectively. The alpha tocopherol standard from Calbiochem (Merck, Darmstadt, Germany) was used. The results were expressed in mg/kg.

### 2.11. Volatile Compounds

Volatile compounds were determined as extensively described by Caipo et al. [33] using 4-methyl-2-pentanol as the internal standard. The volatile compounds were previously equilibrated in a headspace using a HT280T autosampler (HTA s.r.I, Brescia, Italy), automatically controlled by the HT-COMSOFT software (HTA s.r.I), and subsequently adsorbed on a SPME fiber (2 cm length and 50/30 µm film thickness) consisting of a flexible and stable divinylbenzene/carboxene/polydimethylsiloxane (DVB/CAR/PDMS) stationary phase purchased from Supelco (Bellefonte, PA, USA). Then, the volatile compounds were desorbed in the injection port with the purge valve from (splitless mode) a Shimadzu GC-2010 Plus gas chromatograph with a flame ionization detector (FID) (Shimadzu, Kyoto, Japan) using a TR-WAX capillary column (60 m 0.25 mm i.d., 0.25 m coating; Teknokroma, Barcelona, Spain), and a PC with Ver. 2 Workstation Software (Shimadzu, Kyoto, Japan) for data processing. Calibration curves using Sigma standards were made in triplicate with five different concentrations in a range from 6 to 1000 µg/mL with coefficients of determination (R^2^) between 0.857 and 1.000. The results were expressed in mg/kg.

### 2.12. Statistical Analysis

First, a descriptive analysis using ANOVA and Least Square Distance Fisher analysis (LSD-Fisher) methods was performed, in order to detect significative differences between the studied groups. Furthermore, a multivariate analysis was computed: Principal Components Analysis (PCA) and Discriminant Partial Least Square Regression (PLS-DA). The multivariate analysis was based on a NIPALS algorithm (nonlinear iterative partial least squares) [41] using SIMCA-P+ 14 (MKS Umetrics AB, Malmö, Sweden, 2016). All datasets were centered and scaled to unit variance. All the analyses were validated by a full cross-validation routine, minimizing the prediction residual sum of the squares function (PRESS) in order to avoid the overfitting of the models [42].

## 3. Results and Discussion

In this work, −3 °C was applied for 12 h to avoid a dramatic destruction of tissues and to consider a situation in which an oil can be produced without a clear sensory defect. The oil was extracted from the olives with and without treatment, and the analyses were carried out in parallel for a comparative purpose. The olives were extracted from the same field to consider no effect other than the ripening and the freezing treatment.

### 3.1. Quality Parameters

**Figure 1:**
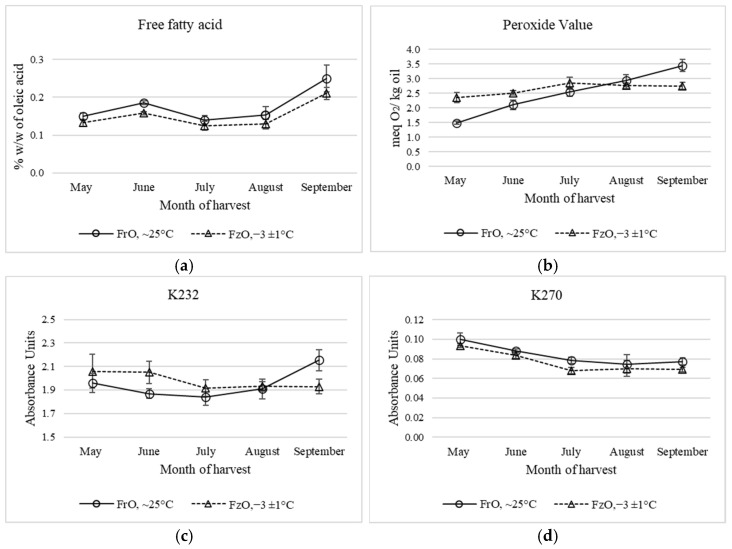
Physicochemical characterization of virgin olive oils extracted from fresh and frozen olives harvested between May and September. Values from (**a**) free fatty acid, (**b**) peroxide value, (**c**) K232, and (**d**) K270 are plotted.

The evolution of quality parameters, including free fatty acids, PV, K232, and K270 measured in Arbequina variety virgin olive oil, issued from fresh and frozen olives at different ripening stages, are reported in Figure 1. All the values of the four parameters measured remained within the limits established by the International Oil Council (IOC) trade standards in the extra virgin olive oil category [8]. When comparing oils obtained from fresh olives with oils obtained from frozen olives, some differences were observed. Free acidity was slightly higher in oils from fresh olives, with significant differences being observed in the month of June. In both types of oils, a significant increase in acidity is observed with fruit ripening or harvesting time, especially in June and September. This difference would be due to the increase in the activity of lipolytic enzymes in the fruit when ripening [43]. The lower acidity value for oils from frozen olives compared with oils from fresh olives is due to the decrease in lipase activity during malaxation, which is caused by freezing of the fruit [43]. Thus, Navajas-Porras et al. [43] reported an increase in acidity level with the ripening of Manzanilla and Picual extra virgin olive oils.

In relation to the peroxide value (PV), a different behavior was observed. Thus, VOOs between May and July showed higher a PV, reaching 2.84 meq O_2_/kg of oil, with no increase in the remaining months. Meanwhile, oils from fresh olives showed a sustained increase in PV with harvest time, reaching a PV of 3.44 meq O_2_/kg oil in September. During the ripening of olives, different metabolic pathways are in motion, prompting several changes in composition that could also affect quality parameters of olive oil [43].

The higher PVs in oils from frozen olives, for the months of May to July, would be related to the green pigmentation of the olives, which decreases as the fruit ripens. Thus, the damage produced in the olives by freezing would affect the chloroplasts of the fruit, leaving the chlorophyll compounds more exposed to light, thus promoting early photoxidation reactions. The light-activated chlorophyll compounds would activate the fundamental oxygen to the singlet state, which would act on the unsaturated fatty acids in the oil, thus producing unstable hydroperoxides [33,44]. The decrease in chlorophyll pigments at advanced stages of fruit ripening would explain the slight, although not significant, decrease in the PVs in oils from frozen olives. Regarding the changes in the PVs when the olives are ripening, there are contradictory results depending on the variety. Whereas for Gemlik, Halhalı, and Picual, PVs in olive varieties were found to increase during ripening, although for the Manzanilla variety, such an index did not change significantly [43,45]. Morello et al. [25] also found a slight rise in PV when the olives were affected by frost.

The K232 absorbance values showed a similar trend to the PV values, with higher K232 values in oils from frozen olives up to July, reversing this behavior in September, with K232 values of 2.15 in oils from fresh olives. Absorbance at 232 nm measures the hydroperoxides of conjugated fatty acids formed in the autoxidation of lipids, and is indicative of primary oil oxidation [46].

The decomposition of the hydroperoxides originates from the secondary oxidation compounds, which are measured at 270 nm [33]. In general, K270 values were low for all the oils evaluated, indicating a raw material in a good state of preservation, despite the effects produced by the freezing of the olives. A greater decomposition of hydroperoxides was observed in oils from fresh olives, giving significantly higher K270 values for the months of July and September. A decrease in K270 values was observed with fruit ripening.

For virgin olive oils, the Manzanilla variety showed no significant changes to K270 when ripening, whereas a significant (*p* < 0.05) decreasing tendency was observed for K232 [43].

### 3.2. Evolution of Color Parameters

**Figure 2:**
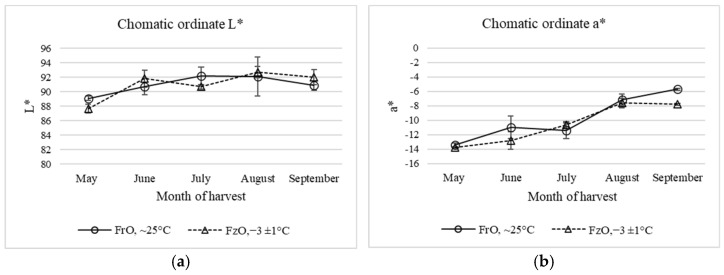

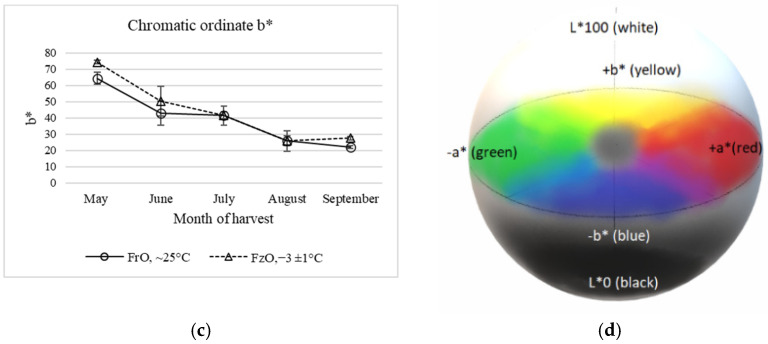
Color expressed as chromatic ordinates (**a**) L*, (**b**) a* and (**c**) b*, in VOO from fresh and frozen olives harvested between May and September. (**d**) Three-dimensional colour space CIELab.

Figure 2 shows the evolution of the chromatic coordinates L, a, and b of oils from fresh and frozen olives. The color of the virgin olive oil samples was determined by chromatic coordinates of CIELAB colorimetric system. In the early ripening stage (May), the oils from fresh and frozen Arbequina olives showed high values of L* Luminosity at 89.10 and 87.65, respectively, negative values of a* at −13.43 and −13.76, respectively, representing medium greenish colors, and positive values of b* of 64.40 and 74.30, respectively, representing yellow colors. These values characterize the Arbequina variety, generally known for its “golden yellow” color, being of a less intense color than other varieties such as Picual, which displays more intense greenish shades [47], due to a lower chlorophyll content compared with other VOO varieties, and other pigments being predominant, such as pheophytins and carotenoids (lutein, β-carotene, violaxanthin, neoxanthin, xanthophylls), which give the yellow color to the oil.

An increase of the lightness parameter (L*) with harvesting time was observed for both types of oils, showing significant differences only at the early ripening stage. The L* values for frozen olive oils were slightly higher in June, August, and September, but were not significant. The chromatic coordinate a* varied between −13.43 and −5.71 and −13.76 and −7.78 for fresh and frozen olive oils, respectively, moving to less green tone, presenting significant differences in the month of September at the advanced ripening stage. The chromatic coordinate b varied between 64.40 and 21.87 and 74.30 and 27.52 for fresh and frozen olive oils, respectively, decreasing the yellow tonality, and showing significant differences between early and advanced ripening stages (*p* < 0.05). Studies have shown that, with the ripening of the fruit, as the harvest months progress, the color of the olive fruit changes from deep green to yellow-green, and then to purple and black, due to the progressive decrease of chlorophyll and carotenoid pigments, which are responsible for the green color of the fruit [47], as the chlorophyll fraction the one that disappears faster than the carotenoid fraction [48]. This gives way to the synthesis of anthocyanin compounds, which appear first as small reddish spots on the skin until it becomes black at full maturation. Only chlorophylls and carotenoids, which are fat-soluble, are transferred to the virgin olive oil [47], giving its characteristic color, which will vary depending on the month of harvest. Morelló et al. [25] observed slight differences in the carotenoid and chlorophyll contents, reflected in higher luminosity values (L*) in oils from olives damaged by frost; however, chromatic ordinates a* and b* were not affected by the low temperatures.

The ΔE values for oils from fresh and frozen olives were 43.26 ± 4.00 and 47.36 ± 1.29, respectively, which points out a greater loss of color in oils from olives damaged by frost. The loss of the characteristic yellowish-green hue of EVOO would be due to the loss of carotenoid and chlorophyll pigments, probably due to the action of chlorophyllase and lipoxygenase enzymes favored by the deterioration of the olive fruit [25]. 

### 3.3. Behavior of Antioxidant Compounds

#### 3.3.1. Phenolic Compounds

Table 1 presents the composition of phenolic compounds in VOO from fresh and frozen Arbequina variety olives. The VOO from fresh olives presented a content of 413 mg/kg of total phenols, higher than that reported by Caipo et al. [33] which had a content of 273 mg/kg, but in the range of other studies [9,49]. In the VOOs from both types of fruit, a significant decrease in phenolic compounds was observed during ripening, with the oleuropein-derived compounds 3,4-DHPEA-EDA and 3,4-DHPEA-EA-AH being the most affected. Romero-Segura et al. [50] reported a decrease of phenolic compounds in VOOs of the Arbequina variety between 20 and 35 weeks after flowering. During olive ripening, the concentration of total phenols progressively increases until it reaches a maximum level at the “cherry” stage, decreasing abruptly as ripening progresses. The rapid decrease of phenolic content during the black maturation phase has relates to a higher activity of hydrolytic enzymes in this period [51].

When comparing the oils obtained from fresh olives with those from frozen olives, it was observed that the latter presented a higher content of total phenols between June and August, and a higher content of 3,4-DHPEA-EDA at almost all harvesting times. This behavior would be due to the early action of the endogenous β-glucosidase enzyme on oleuropein in freeze-damaged olive fruits, which would induce the production of oleuropein-derived aglycones. Romero-Segura et al. [50] reported the highest affinity of β-glucosidase on oleuropein, resulting in the highest hydrolysis level for oleuropein, followed by ligstroside and demethyloleuropein. In other studies, the content of the main phenolic glycosides, oleuropein and demethyloleuropein, in the olive tissue were severely reduced by freezing, and high levels of hydrolytic secoiridoid derivatives (3,4-DHPEA-EDA, 3,4 DHPEA-EA) were found in freeze-damaged fruits [52]. Table 1 further shows an increase in the levels of elenolic acid and hydroxytyrosol in VOOs from freeze-damaged olive fruits as a result of the hydrolytic degradation of oleuropein derivatives. In addition, a decrease in the concentrations of p-HPEA-EDA, and the flavonoids luteolin and apigenin, and an increase in the oxidized fraction of p-HPEA-EDA, were observed. The lower levels of phenols in VOOs from freeze-damaged olive fruits compared with oils from fresh olives would be due to the action of polyphenol oxidase (PPO) and peroxidase (POX) enzymes, which would be released from their sub-cellular compartments by the damage produced during freezing of the fruit and would come into contact with the phenols.

PPO is the main enzyme involved in the oxidation of phenols during physiological processes associated with fruit ripening and during any form of manipulation of the fruit involving tissue damage or breakage. The phenol oxidation by PPO includes two different reactions, hydroxylation of monophenols to form orthodiphenols and the oxidation of orthodiphenols to form o-quinones [53]. POX would also act on orthodiphenols in the presence of H_2_O_2_ to produce semiquinones, o-quinones, and polymers [53,54]. García-Vico et al. [52] described an inactivation of PPO enzymes in olives subjected to freezing for 3 days at −18 °C; however, POX activity remained unaltered, due to the fact that this enzyme is mainly located in the seed, the tissue of which is rather more resistant to adverse environmental conditions. In the same study, as already mentioned, a marked increase of secoiridoid derivatives was observed in the freeze-damaged olive fruits from the Picual and Arbequina varieties, and a decrease of these compounds in the VOOs from these varieties. Guillaume et al. [55] reported a low secoiridoid and high vanillin and vanillic acid content in oils from the Picual trees grown in areas of Australia with recurrent freezing problems. Peres et al. [56] observed higher PPO activity in olive fruits at early ripening stages, and conversely, higher POX activity at more mature stages. In the study carried out by Romero et al. [26] on VOOs with a ‘frostbitten olives’ sensory defect, the authors described a lower concentration of all the phenolic groups, except for secoiridoids, which presented a mean concentration similar to that of extra virgin olive oils.

#### 3.3.2. α-Tocopherol

Table 1 describes the behavior of α-tocopherol in VOOs from fresh and frozen olives at different ripening stages. Virgin olive oils extracted from fresh olives presented a content of 166 mg α-tocopherol/kg oil, which was lower than that reported by Caipo et al. [33] for the same variety. Table 1 also shows a decrease of α-tocopherol, with harvest time and frost condition reaching September levels of 114 and 99 mg/kg α-tocopherol for VOOs from fresh and frozen olives, respectively. Peres et al. [56] reported a decrease of α-tocopherol in the ripening stages of two Portuguese VOO varieties. When comparing the oils of both raw materials, the highest loss of α-tocopherol due to frost condition was observed in the month of May with a 26% loss. In the remaining months, the losses of α-tocopherol fluctuated between 7 and 17%. The stress produced by the freezing of the fruit would promote a series of chemical and enzymatic reactions such as the oxidation of bioactive compounds as phenols, unsaturated fatty acids and pigments [25,26], among others. The major loss in early ripening stages would be due to the green pigmentation of the olives, and thus, to the action of chlorophyll pigments in the cold-damaged olive, producing an early photooxidation of triglycerides as explained above. α-Tocopherol would act by trapping the singlet oxygen, activated by chlorophyll pigments, via a charge transfer attenuation mechanism [57], resulting in the production 8-hydroperoxy-tocopherone, which is transformed into tocopherylquinone in mild acidic conditions with the consequent loss of α-tocopherol [58]. The higher loss of α-tocopherol was related to the higher PV values in VOO in this period (Figure 1); however, Morelló et al. [25] did not observe significant differences in the content of α-tocopherol in VOO obtained from olive fruits harvested after frost damage.

### 3.4. Antioxidant Capacity

The antioxidant capacity of virgin olive oils from fresh and frozen olives is also presented in Table 1. The ORAC assay measures the ability of compounds, in this case phenols, to deactivate radicals by a hydrogen atom transfer mechanism (HAT) [39]. This radical trapping capacity will depend on the composition of the phenol compounds in the oil and their concentration in the medium. A significant decrease (*p* < 0.05) in antioxidant capacity with harvesting time was observed for oils from fresh and frozen olives with ORAC values between 5.2–2.3 and 4.8–2.1 µmol TE/g of oil, respectively. The decrease in antioxidant capacity would be directly related to the significant decrease in phenolic compounds during the ripening of the olives, as already mentioned. Navajas-Porras reported a decrease in the antioxidant capacity of Manzanilla and Picual EVOOs during olive maturation [43]. In addition, significant differences (*p* < 0.05) in antioxidant capacity were observed between both types of oils for the same harvesting time. Oils from frozen olives showed significantly higher antioxidant capacity values between June and August (*p* < 0.05), which could be related to the slightly higher content of total phenols in the case of some particular phenols such as 3,4-DHPEA-EDA and Hydroxityrosol; however, a previous study revealed that when the olives are affected by frost on the tree, the amount of phenols was lower compared with oils from non-frost olives [26]. A direct relationship was observed between total polyphenol content and antioxidant capacity considering 21 measurements (r = 0.9380), which indicates that phenolic compounds can act as effective radical chain-breaking antioxidants. Ballus et al. [59] already reported a high and significant positive correlation between total phenolic contents and the ORAC results (r^2^ = 0.7431; *p* < 0.001).

### 3.5. Volatile Compounds

The composition of volatiles compounds is presented in Table 2. The oil from fresh olives presented a profile of volatile compounds characteristic of a fresh oil, with a marked increase in C6 hexanal, (*E*)-2-hexenal compounds in the first months of harvesting; however, oils from frozen olives showed a lower development of volatile compounds during ripening, especially those generated by the enzymes of the lipoxygenase cascade such as aldehydes and C6 alcohols (hexanal, (*E*)-2-hexenal, hexan-1-ol, (*E*)-2-hexen-1-ol). In fact, for all the harvesting months, a significantly lower content of total volatile compounds was observed in oils from frozen olives compared with the oils from fresh olives (*p* < 0.05). Within each group, no significant differences were observed in the content of total volatile compounds between the different harvesting months (*p* > 0.05); however, significant differences (*p* < 0.05) were observed for some individual compounds (Table 2). The reduced development of volatile compounds in oils from frozen olives is due to a decrease in the activity of the enzymes of the lipoxygenase cascade. Thus, in the study by García-Vico et al. [52], they observed an abrupt drop in the activity of LOX, HPL, ADH, and AAT enzymes in olives damaged by freezing for 3 days at −18 °C. It was also observed that C6 aldehydes were more affected than C6 alcohols, which would result in oils with less bitterness and intensity of green and grassy aroma, but with a higher intensity of fruitiness and with a milder aroma.

In the study carried out by Romero et al. [28] using samples with defective frostbitten olives, the characteristic volatile compounds of two types of frost that affect the composition of the VOO were described. One of them, characterized by ‘soapy’ and ‘strawberry’ perceptions, presented a higher content of ethyl 2-methyl butanoate and ethyl propanoate, whereas the other, characterized by the ‘wood’ and ‘humidity’ descriptors, presented a high concentration of pentanal and octanal; however, in our study, higher levels of these compounds were not observed in oils from frozen olives. This observation could be explained by the fact that the olives were affected by mild frost (−3 °C) instead of a severe frost, and therefore, the volatile composition did not change so much as to produce the characteristic volatile compounds of defective frostbitten olives.

### 3.6. Multivariate Characterization of Samples Using PCA and PLS-DA

**Figure 3:**
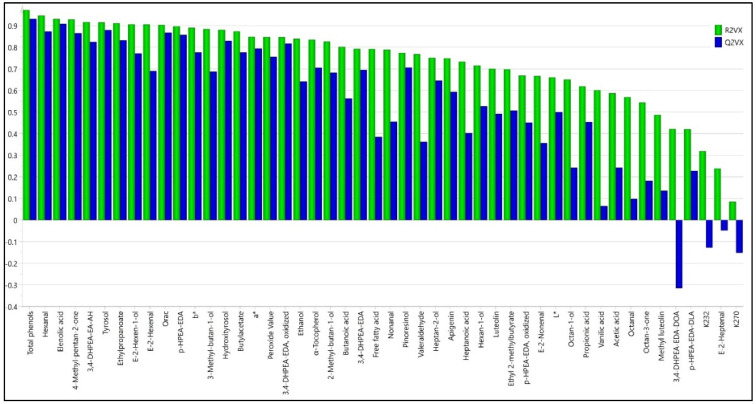
Explained fraction (R^2^VX) of the variation of the X variables (48 phenolic and volatile compounds and physical–chemical parameters) obtained from the virgin olive oil samples.

Given that the olives were affected by mild frost instead of severe frost and some univariate statistical differences were observed, the next step was to evaluate if the temperature of −3 °C during 12 h caused enough differences in the composition of phenols and volatile compounds, and other quality parameters, to be observable in a multivariate statistical analysis. Thus, for this purpose, the data were analyzed by principal component analysis (PCA). These consisted of 48 compounds and quality indexes (variables). As a first approach, the most informative variables classifying oils from fresh and frozen olives were selected by considering the model contribution and residuals of the variables (R^2^VX). The residuals inform the extent to which each variable is modelled. Thus, R^2^VX represents the fraction of X variation which is modeled in the component [60], ranging from 0 to 1, and a R^2^VX < 0.5 was considered as selection criterion to select those compounds (variables) that are well explained. Figure 3 shows the R^2^VX for each compound. The initial reproducibility/prediction (Q^2^VX) was also shown. This parameter indicates the fraction of the variation of a variable that can be predicted by a component as estimated by cross-validation [61]; however, in this case, only R^2^VX accounted for selecting variables, since this was a first descriptive approximation, and prediction performance was studied later. Thus, considering the explained variation, the following variables were removed for having R^2^VX < 0.5 (Figure 3): methyl luteoline, aglycone of oleoeuropein dialdehidic, aglycone of ligustroside dialdehidic, K232, and (*E*)-2-heptenal, K270.

**Figure 4:**
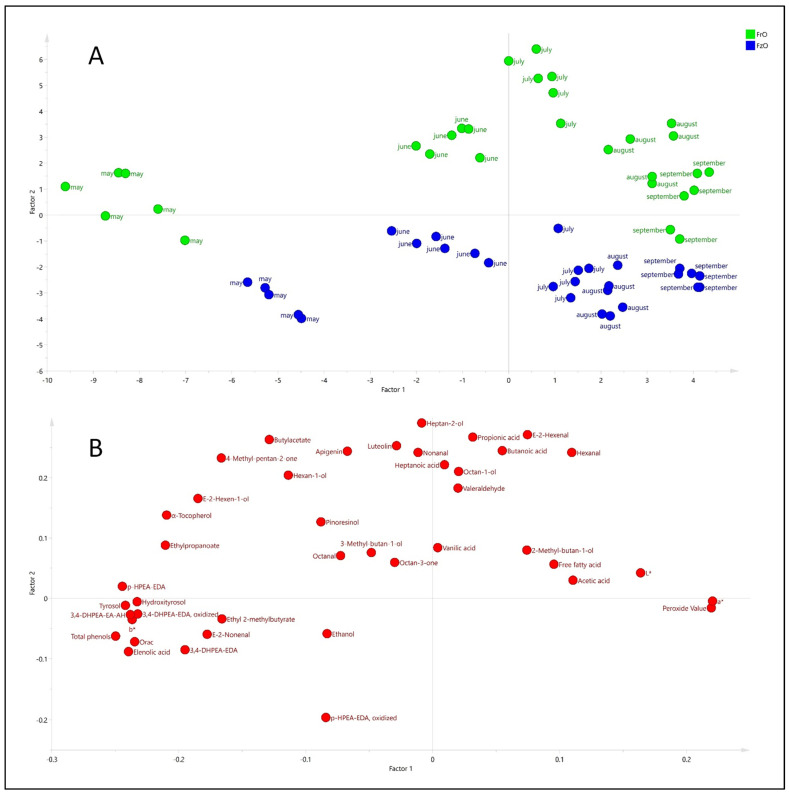
Results of principal component analysis (PCA) carried out with 42 variables. The scores (**A**) and loading (**B**) plots are shown, and virgin olive oils extracted from fresh (FrO) and frozen (FzO) olives are highlighted with different color.

Once the 6 compounds mentioned above were omitted, the PCA was repeated. The result pointed out that 80.2% of the total variance was explained with 5 factors having an explained variance of 35.2% (F1), 19.1% (F2), 12.9% (F3), 7.03% (F4), and 5.9% (F5). Figure 4 shows the scores and loading plots for F1 and F2. Factor 2 showed a separation of the samples from fresh and frozen olives. The direct (or inverse) contribution of variables and observations could be associated by superimposing the hyperplanes of scores. Thus, the samples from the fresh olives (in green color) had a direct relationship with the variables in the upper quadrants, compared with those treated at −3 °C (in blue color). The comparison of scores and loading plots shows the influence of the phenols (e.g., tyrosol, hydroxityrosol, dialdehydic form of decarboxymethyl ligstroside aglycon, dialdehydic form of decarboxymethyl oleuropein aglycon and aldehyde, and hydroxylic form of oleuropein aglycone) in the samples from fresh olives in the early season (May) and the contribution of peroxide value in the advanced ripening season. The influence of volatile compounds characterizing oils from fresh olives compared with frozen olives was also observed.

**Figure 5:**
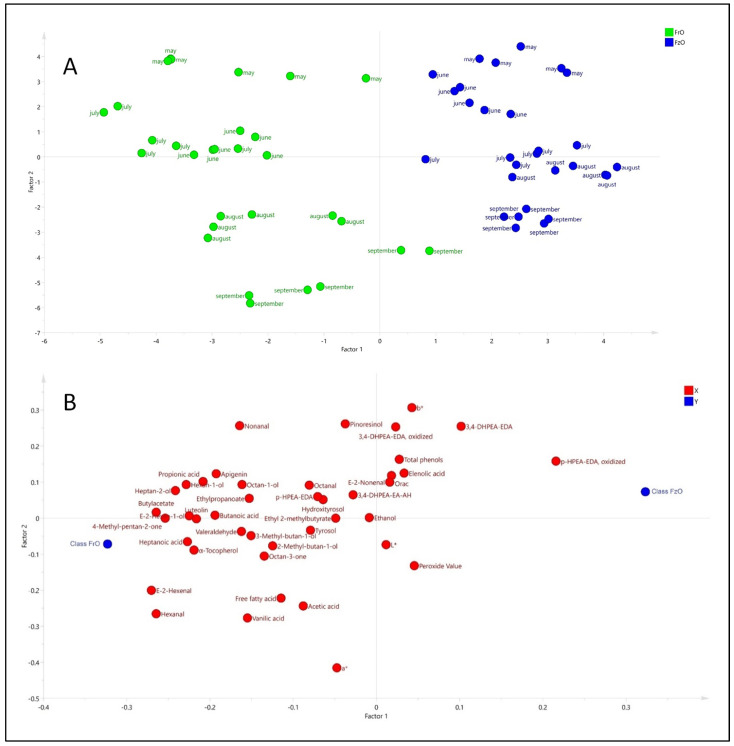
Results discrimination of partial least square (PLS-DA) analysis carried out on the selected variables to classify virgin olive oils from fresh (FrO) and frozen (FzO) olives (**A**) and the corresponding loadings plot (**B**).

Considering the differences in composition and quality characteristics between oils from fresh and frozen olives during ripening, PLS-DA was applied. Figure 5 shows the results of this analysis. The PLS-DA model retained 4 factors, explaining 94.4% (R^2^Y) of the variance (discrimination between the two groups) and a validity/reproducibility of 90.1% (Q^2^). Thus, the groups were clearly separated according to the temperature treatment of the olives. The PLS-DA also allowed a separation of oils from fresh and frozen olives, and an evolution during ripening was also observed. Thus, the ripening effect was observed in factor 2, whereas factor 1 permitted a separation of the two types of samples. The loading plot, also shown in Figure 5, proved the contribution of phenols and volatile compounds for the separation of oils from fresh olives as well; however, the relative importance of phenols for clustering samples from the early stages of ripening (May) was not so clear as in the case of PCA (Figure 4).

## 4. Conclusions

The results proved that the temperature associated with mild frost, −3 °C, over 12 h, produced a clear effect on the composition of phenols, volatile compounds, and quality parameters. Thus, this temperature may permit the production of virgin olive oils with no defects, although some quality decay is expected, which is reflected in the lower concentration of volatile compounds.

PCA and PLS-DA analysis clearly differentiated between VOOs from fresh olives and VOOs from frozen olives, showing that mild frost also promotes a series of biochemical reactions in the fruit that affects the final VOO composition. In addition, these statistical analyses carried out on the physical chemical and chemical data of the samples also showed a separation of oils according to their harvest time. Thus, this result points out that fruit ripening is another important variable to consider at the time of olive harvesting, particularly when VOOs with high levels of phenolic compounds are desired. The frost condition makes the harvesting time more relevant, especially in relation to α-tocopherol, which mainly declined in oils from frozen olives. Although the total concentration of volatile compounds was not affected by harvest time, there was less development in oils from frozen olives, particularly aldehydes and C6 alcohols related to the lipoxygenase cascade (hexanal, (*E*)-2-hexenal, hexan-1-ol, (*E*)-2-hexen-1-ol).

Oils from olives subjected to mild frost preserved the basic characteristics of virgin olive oil, with minor changes in their chemical quality parameters and with a greater impact on volatile compounds, while maintaining the phenolic compounds responsible for the healthy properties of the oil.

As a general conclusion, the effect of freezing at a mild temperature (−3 °C) was influenced by ripening, although no general conclusion can be achieved for all the chemical and physical–chemical parameters. This information complements previous studies and highlights the importance of considering freezing phenomena to have efficient harvesting optimization and control quality, particularly in areas with a high risk of low temperatures.

## Figures and Tables

**Table 1:** Effect of frost and time of harvest in the composition of phenols, α-tocopherol and antioxidant capacity of Arbequina VOO.

	Months of Harvest									
Phenolic Compound (mg/kg)	May		June		July		August		September	
	FrO	FzO	FrO	FzO	FrO	FzO	FrO	FzO	FrO	FzO
Elenolic acid	231 ± 6^Aa^	226 ± 14^Aa^	93 ± 9^Ab^	116 ± 6^Bb^	74 ± 3^Ac^	113 ± 6^Bb^	66 ± 3^Ad^	73 ± 3^Bc^	64 ± 8^Ad^	68 ± 5^Ac^
Hydroxityrosol	3.8 ± 0.6^Aa^	1.2 ± 0.2^aB^	0.89 ± 0.08^Ab^	1.6 ± 0.2^Bb^	0.23 ± 0.01^Ac^	0.28 ± 0.04^Bc^	0.20 ± 0.02^Ac^	0.43 ± 0.04^Bd^	0.21 ± 0.04^Ac^	0.08 ± 0.02^Be^
Tyrosol	2.3 ± 0.2^Aa^	1.3 ± 0.1^Ba^	0.99 ± 0.151^Ab^	0.8 ± 0.1^Bb^	0.31 ± 0.03^Acd^	0.33 ± 0.06^Ac^	0.22 ± 0.04^Ac^	0.34 ± 0.05^Bc^	0.42 ± 0.05^Ad^	0.27 ± 0.02^Bd^
Vanilic acid	0.07 ± 0.01^Aa^	0.09 ± 0.0^Ba^	0.06 ± 0.01^Ab^	0.06 ± 0.01^Ab^	0.09 ± 0.01^Ac^	0.07 ± 0.01^Bc^	0.09 ± 0.01^Aac^	0.07 ± 0.01^Bc^	0.12 ± 0.03^Ad^	0.05 ± 0.01^Bd^
p-Coumaric acid	ND	ND	ND	ND	ND	ND	ND	ND	ND	ND
*3,4-DHPEA-EDA*	68 ± 5^Aa^	85 ± 8^Ba^	65 ± 9^Aa^	89 ± 4^Ba^	31 ± 2^Ab^	49 ± 5^Bb^	9 ± 1^Ac^	50 ± 3^Bb^	23 ± 2^Ad^	0.46 ± 0.01^Bc^
*3,4-DHPEA-EDA,* oxidized	3.0 ± 0.2^Aa^	3.0 ± 0.2^Aa^	1.0 ± 0.1^Ab^	2.7 ± 0.2^Bb^	1.1 ± 0.1^Ac^	0.1 ± 0.0^Bc^	0.1 ± 0.0^Ad^	0.4 ± 0.0^Bd^	0.1 ± 0.0^d^	ND
*3,4-DHPEA-EDA-DOA*	0.07 ± 0.00^a^	ND	0.09 ± 0.02^Ab^	0.05 ± 0.00^Ba^	0.05 ± 0.01^Ac^	0.08 ± 0.01^Ba^	0.06 ± 0.01^Aac^	0.06 ± 0.01^Aa^	0.10 ± 0.02^Ad^	0.03 ± 0.00^Ba^
*p-HPEA-EDA*	50 ± 2^Aa^	43 ± 2^Ba^	39 ± 1^Ab^	29 ± 2^Bb^	21 ± 1^Ac^	20 ± 1^Bc^	21 ± 2^Ac^	23 ± 2^Ad^	12 ± 2^Ad^	11 ± 1^Ae^
*p-HPEA-EDA,* oxidized	0.33 ± 0.02^Aa^	1.37 ± 0.27^Ba^	0.30 ± 0.04^Ab^	0.88 ± 0.09^Bb^	0.06 ± 0.01^Ac^	0.19 ± 0.01^Bc^	0.13 ± 0.01^Ad^	1.29 ± 0.12^Ba^	0.16 ± 0.02^e^	ND
Pinoresinol^1^	9 ± 1^Aa^	8.8 ± 0.4^Aa^	12 ± 1^Ab^	11 ± 0^Bb^	12 ± 1^Ab^	8.5 ± 0.3^Ba^	8 ± 1^Ac^	9 ± 1^Ba^	6.9 ± 0.3^Ac^	7 ± 1^Ac^
*p-HPEA-EDA-DLA*	1.8 ± 0.1^Aa^	2.6 ± 2.0^Aa^	1.6 ± 0.1^Ab^	1.7 ± 0.1^Ab^	1.6 ± 0.1^Ab^	0.6 ± 0.1^Bc^	0.8 ± 0.1^Ac^	1.1 ± 0.1^Bc^	0.9 ± 0.1^Ac^	0.8 ± 0.1^Bc^
*3,4-DHPEA-EA-AH*	30 ± 2^Aa^	36 ± 2^Ba^	13 ± 3^Ab^	14 ± 1^Ab^	10 ± 1^Ac^	2 ± 0^Bc^	6 ± 1^Ad^	7 ± 1^Bd^	5 ± 0^Ad^	0.3 ± 0.0^Be^
Luteolin	8.0 ± 0.6^Aa^	6.6 ± 1.5^Ba^	8.9 ± 0.8^Aa^	8.5 ± 0.3^Ab^	14 ± 2^Ab^	5.4 ± 0.2^Bc^	8.8 ± 2.2^Aa^	8 ± 1^Bb^	8 ± 1^Aa^	3.4 ± 0.3^Bd^
Apigenin	3.1 ± 0.3^Aa^	2.5 ± 0.4^Ba^	3.1 ± 0.3^Aac^	2.8 ± 0.2^Ab^	4.5 ± 0.7^Ab^	2.4 ± 0.2^Ba^	2.7 ± 0.5^Acd^	2.4 ± 0.5^Aa^	2.6 ± 0.3^Ad^	1.6 ± 0.3^Bc^
Methyl luteolin	1.6 ± 0.1^Aa^	1.7 ±0.7^Aab^	2.0 ± 0.3^Ab^	1.8 ± 0.2^Aa^	2.0 ± 0.2^Ab^	1.8 ± 0.2^Aa^	1.3 ± 0.3^Ac^	1.3 ± 0.2^Abc^	1.4 ± 0.4^Aac^	1.0 ± 0.3^Bc^
Total phenols	413 ± 7^Aa^	419 ± 10^Aa^	241 ± 6^Ab^	279 ± 10^Bb^	171 ± 5^Ac^	204 ± 9^Bc^	124 ± 3^Ad^	177 ± 8^Bd^	124 ± 8^Ad^	93 ± 5^Be^
non-oxidized phenols	410 ± 7	415 ± 10	240 ± 6	276 ± 10	170 ± 5	204 ± 9	124 ± 3	175 ± 8	124 ± 8	93 ± 5
Oxidized phenols	3.40 ± 0.16	4.41 ± 0.34	1.25 ± 0.06	3.60 ± 0.22	1.15 ± 6	0.23 ± 0.01	0.24 ± 0.02	1.74 ± 0.16	0.26 ± 0.02	ND
α-Tocopherol (mg/kg)	166 ± 8^Aa^	123 ± 3^Ba^	131 ± 4^Ab^	115 ± 6^Bb^	125 ± 7^Ab^	114 ± 2^Bb^	117 ± 5^Ac^	97 ± 6^Bc^	114 ± 1^Ac^	99 ± 5^Bc^
ORAC (µmol TE/g)	5.2 ± 0.6^Aa^	4.8 ± 0.2^Ba^	2.9 ± 0.2^Ab^	3.6 ± 0.7^Bb^	2.4 ± 0.1^Ac^	2.9 ± 0.2^Bc^	2.9 ± 0.1^Ad^	3.0 ± 0.1^Ac^	2.3 ± 0.1^Ac^	2.1 ± 0.3^Bd^

Values are mean ± SD (*n* = 3). Different capital letters in the same row for each month indicate significant differences between VOO from fresh and frozen olives (*p* < 0.05; LSD Fisher). Different lower-case letters in the same row indicate significant differences between oils of different ripening stages (*p* < 0.05; LSD Fisher). Oils from fresh and frozen olives are compared separately. Abbreviations: FrO, Oil from fresh olives. FzO, oil from frozen olives. ^1^: Mixed with 1-acetoxy-pinoresinol. 3,4-DHPEA-EDA, dialdehydic form of decarboxymethyl oleuropein aglycon. 3,4-DHPEA-EDA-DOA, dialdehydic form of oleouropein aglycon. p-HPEA-EDA, dialdehydic form of decarboxymethyl ligstroside aglycon. p-HPEA-EDA-DLA, dialdehydic form of ligstroside aglycon. 3,4-DHPEA-EA-AH, aldehyde, and hydroxylic form of oleuropein aglycone. ND, not detected.

**Table 2:** Effect of frost and time of harvest in the composition of volatile compounds in the VOO Arbequina variety.

	Months of Harvest											
	May		June		July		August		September			
Volatile Compounds (mg/kg)	FrO	FzO	FrO	FzO	FrO	FzO	FrO	FzO	FrO	FzO	Sensory Atributes	OT in Oil (mg/kg)
Ethanol	6.61 ± 4.64^Aa^	1.47 ± 0.45^Ba^	0.51 ± 0.03^Ab^	0.81 ± 0.06^Ba^	1.04 ± 0.50^Ab^	3.04 ± 1.64^Bbc^	2.46 ± 0.41^Ab^	3.77 ± 0.58^Bc^	1.87 ± 0.30^Ab^	2.78 ± 0.75^Bb^	Apple, sweet	30
Ethylpropanoate	0.14 ± 0.02^Aa^	0.08 ± 0.01^Ba^	0.09 ± 0.00^Ab^	0.08 ± 0.00^Ba^	0.08 ± 0.01^Ac^	0.08 ± 0.01^Aa^	0.08 ± 0.01^Ac^	0.07 ± 0.01^Ab^	0.07 ± 0.00^Ac^	0.06 ± 0.00^Bb^	Strawberry, apple, fruity	0.10
Pentanal	0.09 ± 0.02^Aa^	0.08 ± 0.01^Aa^	0.25 ± 0.04^Ab^	0.17 ± 0.08^Bb^	0.15 ± 0.02^Ac^	0.08 ± 0.01^Bac^	0.12 ± 0.02^Ad^	0.06 ± 0.03^Ba^	0.16 ± 0.02^Ac^	0.13 ± 0.07^Bbc^	Woody, bitter, oily	0.24
4-Methyl-pentan-2-one	0.09 ± 0.01^Aa^	0.07 ± 0.01^Ba^	0.10 ± 0.00^Ab^	0.06 ± 0.00^Ba^	0.09 ± 0.01^Ac^	0.04 ± 0.01^Bb^	0.06 ± 0.00^Ad^	0.03 ± 0.01^Bb^	0.05 ± 0.00^Ae^	0.04 ± 0.00^Bb^	Strawberry, fruity, sweet, ethereal	0.30
Ethyl 2-methylbutyrate	0.03 ± 0.01^Aa^	0.02 ± 0.00^Ba^	0.02 ± 0.01^Ab^	0.02 ± 0.00^Aa^	0.01 ± 0.00^Ab^	0.02 ± 0.01^Ba^	0.02 ± 0.01^Ab^	0.02 ± 0.00^Aa^	0.02 ± 0.00^Ab^	0.02 ± 0.00^Aa^	Fruity	0.72
Butylacetate	0.07 ± 0.01^Aa^	0.04 ± 0.01^Ba^	0.06 ± 0.00^Aa^	0.05 ± 0.00^Ab^	0.07 ± 0.01^Aa^	0.04 ± 0.01^Bad^	0.05 ± 0.00^Ab^	0.03 ± 0.00^Bc^	0.04 ± 0.00^Ac^	0.03 ± 0.00^Bcd^	Green, fruity, pungent, sweet	0.10
Hexanal	4.28 ± 0.38^Aa^	4.49 ± 0.24^Aa^	6.98 ± 0.16^Ab^	4.71 ± 0.12^Bab^	6.19 ± 0.31^Ac^	4.38 ± 0.50^Ba^	6.35 ± 0.57^Ac^	4.66 ± 0.41^Bab^	7.21 ± 0.89^Ab^	5.02 ± 0.34^Bb^	Green apple, grass	0.08
2-Methyl-butan-1-ol	0.07 ± 0.03^Aab^	0.03 ± 0.00^Ba^	0.05 ± 0.01^Aa^	0.05 ± 0.01^Ab^	0.05 ± 0.00^Aa^	0.06 ± 0.01^Ab^	0.07 ± 0.02^Aab^	0.05 ± 0.00^Bb^	0.08 ± 0.02^Ab^	0.07 ± 0.01^Ac^	Winey, spicy	0.48
3-Methyl-butan-1-ol	0.18 ± 0.12^Aa^	0.04 ± 0.01^Ba^	0.04 ± 0.01^Ab^	0.04 ± 0.01^Aa^	0.07 ± 0.01^Abc^	0.07 ± 0.02^Ab^	0.11 ± 0.10^Abd^	0.06 ± 0.01^Ab^	0.12 ± 0.09^Aad^	0.07 ± 0.01^Ab^	Woody, sweet	0.10
(*E*)-2-Hexenal	6.43 ± 0.98^Aa^	6.42 ± 0.47^Aa^	11.07 ± 0.40^Ab^	7.31 ± 0.28^Bb^	9.56 ± 0.88^Ac^	6.32 ± 1.02^Ba^	9.58 ± 0.79^Ac^	6.58 ± 0.69^Bab^	9.38 ± 0.94^Ac^	7.07 ± 0.62^Bab^	Bitter almonds, green- fruity	0.42
Octan-3-one	0.20 ± 0.01^Aa^	0.19 ± 0.00^Ba^	0.19 ± 0.00^Ab^	0.19 ± 0.00^Aa^	0.19 ± 0.00^Ab^	0.19 ± 0.00^Aa^	0.19 ± 0.00^Ab^	0.19 ± 0.00^Aa^	0.20 ± 0.00^Aa^	0.19 ± 0.00^Aa^	Pungent, resinous	0.75
Octanal	0.06 ± 0.01^Aa^	0.09±0.02^Ba^	0.06 ± 0.00^Aa^	0.05 ± 0.01^Ab^	0.06 ± 0.00^Aa^	0.04 ± 0.01^Bb^	0.07 ± 0.02^Aab^	0.05 ± 0.01^Bb^	0.06 ± 0.01^Aa^	0.05 ± 0.01^Ab^	Fatty, Sharp	0.32
(*E*)-2-heptenal	0.59 ± 0.29^Aa^	0.53 ± 0.02^Aa^	0.66 ± 0.16^Aa^	0.65 ± 0.13^Aab^	0.91 ± 0.15^Ab^	0.63 ± 0.12^Bab^	0.52 ± 0.11^Aa^	0.55 ± 0.23^Aab^	0.51 ± 0.17^Aa^	0.74 ± 0.03^Bb^	Sweet, grassy, woody	5.00 × 10^−3^
Heptan-2-ol	2.28 ± 0.35^Aa^	0.96 ± 0.52^Bac^	3.90 ± 0.62^Ab^	3.38 ± 0.12^Ab^	4.49 ± 0.69^Ab^	0,62 ± 0,77^Ba^	3.79 ± 0.85^Ab^	0,59 ± 0,81^Ba^	2.05 ± 0.62^Aa^	1.62 ± 0.81^Ac^	Earthy, sweety	0.01
Hexan-1-ol	5.29 ± 0.91^Aa^	3.94 ± 0.15^Ba^	5.07 ± 0.12^Aa^	3.81 ± 0.06^Bb^	4.72 ± 0.71^Aab^	4.24 ± 0.23^Ac^	4.06 ± 0.47^Ab^	3.79 ± 0.04^Ad^	4.37 ± 0.43^Ab^	4.00 ± 0.28^Abd^	Fruity, soft, aromatic	0.40
Nonanal	0.92 ± 0.14^Aa^	1.09 ± 0.06^Ba^	0.63 ± 0.02^Ab^	0.61 ± 0.06^Abc^	0.63 ± 0.04^Ab^	0.56 ± 0.02^Bb^	0,66 ± 0.03^Ab^	0.65 ± 0.01^Adc^	0.69 ± 0.04^Ab^	0.71 ± 0.05^Ad^	Fatty, waxy, pungent	0.15
(*E*)-2-nonenal	1.08 ± 0.23^Aa^	1.21 ± 0.34^Aa^	0.79 ± 0.09^Aa^	0.72 ± 0.05^Ab^	1.05 ± 0.21^Aa^	0.78 ± 0.14^Ab^	1.92 ± 0.83^Ab^	0.74 ± 0.04^Bb^	1.10 ± 0.25^Aa^	1.07 ± 0.07^Ab^	Fatty, rancid, paper-like, penetrating, waxy, beany	0.90
*(E)*-2-Hexen-1-ol	4.93 ± 0.19^Aa^	4.30 ± 0.05^Ba^	4.65 ± 0.16^Ab^	4.34 ± 0.00^Bb^	4.44 ± 0.07^Ac^	4.24 ± 0.03^Bb^	4.33 ± 0.05^Ac^	4.19 ± 0.03^Bb^	4.30 ± 0.04^Ac^	4.27 ± 0.03^Aa^	Green grass, leaves, fruity, astringent, bitter	5.00
Acetic acid	1.73 ± 0.10^Aa^	1.78 ± 0.04^Aa^	1.72 ± 0.03^Aa^	1.67 ± 0.03^Aa^	1.72 ± 0.03^Aa^	2.02 ± 0.38^Bbc^	2.68 ± 0.93^Ab^	2.10 ± 0.15^Ac^	2.72 ± 0.70^Ab^	1.85 ± 0.08^Bab^	Sour, vinegary	0.50
Propionic acid	0.30 ± 0.02^Aa^	0.29 ± 0.01^Aac^	0.34 ± 0.02^Ab^	0.30 ± 0.00^Bab^	0.34 ± 0.01^Ab^	0.30 ± 0.01^Bb^	0.34 ± 0.03^Ab^	0.30 ± 0.01^Bb^	0.30 ± 0.03^Aa^	0.28 ± 0.01^Ac^	Pungent, sour, mould	0.72
Octan-1-ol	0.41 ± 0.00^Aa^	0.41 ± 0.00^Aab^	0.41 ± 0.01^Aa^	0.41 ± 0.00^Aab^	0.44 ± 0.00^Ab^	0.41 ± 0.00^Bb^	0.43 ± 0.03^Ab^	0.40 ± 0.00^Ba^	0.41 ± 0.00^Aa^	0.41 ± 0.01^Aab^	Green, fusty, musty, sweet, waxy	0.48
Butanoic acid	0.31 ± 0.03^Aa^	0.31 ± 0.01^Aac^	0.32 ± 0.01^Aa^	0.29 ± 0.00^Bb^	0.46 ± 0.03^Ab^	0.32 ± 0.01^Bc^	0.44 ± 0.04^Ab^	0.30 ± 0.00^Bab^	0.31 ± 0.01^Aa^	0.31 ± 0.02^Aabc^	Rancid, cheese, sweat	0.65
Heptanoic acid	2.31 ± 0.09^Aab^	2.27 ± 0.01^Aa^	2.26 ± 0.01^Aa^	2.22 ± 0.01^Bb^	2.36 ± 0.08^Abc^	2.25 ± 0.01^Bac^	2.40 ± 0.02^Ac^	2.24 ± 0.03^Bbc^	2.28 ± 0.05^Aa^	2.25 ± 0.02^Aac^	Rancid, fatty	0.1
Total Volatiles	38.4 ± 2.2^Aa^	30.1 ± 1.7^Ba^	40.2 ± 2.8^Aa^	31.9 ± 2.0^Bab^	39.1 ± 2.5^Aa^	30.7 ± 1.8^Ba^	40.7 ± 2.5^Aa^	31.4 ± 1.9^Bab^	38.3 ± 2.5^Aa^	33.0 ± 1.9^Bb^		

Values are mean ± SD (*n* = 3). Different capital letters in the same row for each month indicate significant differences between VOO from fresh and frozen olives (*p* < 0.05; LSD Fisher). Different lower-case letters in the same row indicate significant differences between oils of different ripening stages (*p* < 0.05; LSD Fisher). Abbreviations: FrO, Oil from fresh olives. FzO, oil from frozen olives. OT, Odour Threshold.

## Data Availability

Data is contained within the article.
